# Sensory Loss and Risk of Dementia

**DOI:** 10.1177/10738584221126090

**Published:** 2022-09-28

**Authors:** Meher Lad, William Sedley, Timothy D. Griffiths

**Affiliations:** 1 Translational and Clinical Research Institute, Newcastle University, Newcastle upon Tyne, UK; 2 Biosciences Institute, Newcastle University, Newcastle upon Tyne, UK; 3 Wellcome Centre for Human Neuroimaging, University College London, London, UK; 4 Human Brain Research Laboratory, University of Iowa, Iowa City, IA, USA

**Keywords:** sensory loss, dementia, olfactory loss, hearing loss, visual loss, cognitive, neurology, Alzheimer disease

## Abstract

Sensory loss in olfaction, vision, and hearing is a risk factor for dementia, but the reasons for this are unclear. This review presents the neurobiological evidence linking each sensory modality to specific dementias and explores the potential mechanisms underlying this. Olfactory deficits can be linked to direct neuropathologic changes in the olfactory system due to Alzheimer disease and Parkinson disease, and may be a marker of disease severity. Visual deficits potentially increase dementia risk in a vulnerable individual by reducing resilience to dementia. Hearing deficits may indicate a susceptibility to Alzheimer disease through a variety of mechanisms. More generally, sensory impairment could be related to factors associated with resilience against dementia. Further research is needed to tease out the specific and synergistic effects of sensory impairment. Studying sensory loss in relation to neurodegenerative biomarkers is necessary to clarify the mechanisms involved. This could produce new monitoring and management strategies for people at risk of dementia.

## Introduction

There is a strong link between sensory loss in midlife and subsequent dementia. Olfactory, visual, and hearing impairments are all associated with an increased risk of subsequent dementia (Dintica and others 2019; Livingston and others 2020; Tran and others 2020). The reasons for this are unclear, but the link is strong and not due to the simple co-occurrence of two common conditions in aging: sensory loss and dementia. The importance of clarifying the nature of this relationship cannot be overstated, as the burden of cognitive impairment and dementia in an aging population worldwide is increasing. Currently, dementia has no treatment or cure, and understanding this relationship could pave the way for novel management strategies based on sensory manipulation for millions of people worldwide.

The evidence for the association between sensory loss and dementia has been assessed separately for each sensory modality. Prospective population-based studies examine the development of dementia in subjects with and without sensory loss, while retrospective cohort studies are based on the examination of sensory impairment in those with and without dementia (Dintica and others 2019; Livingston and others 2020; Tran and others 2020). In some cases, there is consensus on the test used to identify sensory loss, such as using the pure tone audiogram (PTA) as a standard measure of hearing. In other modalities, the definition of *sensory* varies substantially. In vision, the tests range from visual acuity to contrast sensitivity, and in olfaction, the type of odor identification task varies (Dintica and others 2019; Ikram and others 2012; Livingston and others 2020; Quarmley and others 2017; Swenor and others 2019; Wheeler and Murphy 2021).

As evidence grows, more questions emerge: Are one or more modalities privileged, or is there a domain-general risk increase for dementia caused by sensory loss? Does dementia affect sensation, or does sensory loss lead to an increased risk of dementia? Does sensory loss alter the neurobiological process that increases the risk for dementia? In other words, is there a direct interaction between sensory loss and the neurodegenerative disease processes? How does treatment of sensory loss affect neurodegeneration and dementia risk? How can we account for comorbidities of sensory loss, such as mood disturbance and social isolation?

This review summarizes existing evidence for the risk of dementia due to acquired sensory loss in the olfactory, visual, and hearing domains. Certain dementia syndromes involve cortical visual or auditory loss as the primary manifestation. These are mentioned briefly but are not the focus of this review. Human studies are emphasized that shed light on potential mechanisms that may underlie some of the associations with the various sensory modalities, in addition to evidence from the animal work that allows systematic examination of pathologic processes. We focus on Alzheimer disease (AD) as a prototypical dementia where a neurobiological definition of the disease process has enriched our understanding, and there are studies emerging where sensory loss has been examined alongside specific neurodegenerative disease markers. We consider other forms of dementia with distinct cognitive phenotypes and underlying pathology. We cover neuropsychological, neuroimaging, neurochemical, and neuropathologic work that may shed light on mechanisms, and we speculate whether links between sensory loss and dementia are causative. We explore domain-specific models based on sensory deprivation within specific modalities and domain-general models involving cognitive resilience. Finally, we consider future studies that might increase our understanding of the link between sensory loss and risk of dementia.

## The Complex Relationship between Symptoms and Neurodegenerative Markers in Dementia

The term *dementia* refers to a syndrome where a person has cognitive deficits and loses the ability to function independently in his or her daily life. It is most commonly caused by neurodegenerative diseases such as AD, vascular disease (VaD), and Lewy body disease (LBD). Each neurodegenerative dementia shows classical neuropathologic appearances in the brain postmortem, which have been studied extensively, but a particular dementia syndrome could be associated with different pathologies. Frontotemporal dementia, for example, can be associated with neuropathologic proteins in the brain, such as tau, TDP-43, and ubiquitin. We now know that these disease processes, where these proteins are deposited in the brain, may begin in a person’s brain decades before they develop dementia, and in this period one may show subtle cognitive signs of a neurodegenerative disease. Several terms have been associated with this period, such as mild cognitive impairment (MCI), where an individual may show mild cognitive deficits, or a prodromal phase, where there may be no symptoms but neurodegenerative biomarker evidence of disease in the brain.

The diagnosis of the most common type of dementia, AD dementia, is based on clinical criteria with support from neuropsychological, neuroimaging, and biochemical tests (Jack and others 2018). The first classification of AD was based on clinical criteria alone, with impairment of episodic memory signaling the start of the disease. In 2011, revised criteria considered the use of biomarkers, as data began to emerge about the presence of neuropathology relevant to AD across the spectrum of life, from healthy people to those with AD dementia (McKhann and others 2011). As fluid biomarkers became more refined and novel PET brain tracers emerged, these criteria have become more tightly associated with the disease process itself and can now predict conversion from an MCI state to AD dementia with a high degree of accuracy (Palmqvist and others 2021). Although the neuropathologic process can be detected accurately with such biomarkers, the clinical phenotype can indeed be variable; other phenotypes where visual, language, or behavioral symptoms predominate are well recognized. Recent advances in the measurement of neurodegenerative markers specific to AD in vivo have allowed us to measure key proteins, such as amyloid and tau, and stratify a person’s risk of future dementia (Jack and others 2018).

The diagnosis of other dementias remains largely clinical, and the underlying disease process is inferred from the dementia syndrome that a person exhibits. Frontotemporal dementia commonly presents with abnormal behavior and atrophy in frontal and temporal areas as shown by MRI (Snowden and others 2012). As mentioned previously, the underlying pathology can vary among TDP-43 (types A, B, C), tau proteins, and ubiquitin. As yet, there are no good in vivo neurodegenerative markers for these proteins that are specific to neurodegeneration in frontotemporal dementia. Dementia with Lewy bodies usually causes fluctuations in attention, hallucinations, visuospatial ability, and motor symptoms similar to Parkinson disease (PD), either before the motor symptoms or within 1 y of the onset of motor symptoms (McKeith and others 2017). Neuroimaging with a dopamine transporter scan can support the diagnosis of this condition if other dementia syndromes are being considered at the time, but it is not widely considered necessary for the diagnosis of more clinically obvious cases. This condition is neuropathologically typified by the presence of aggregates of α-synuclein in the brain. Recent evidence suggests that fluid biomarkers that detect these proteins are useful indicators of early stages of the LBD (Rossi and others 2021).

Vascular dementia is often diagnosed with clear evidence of VaD burden on MRI with a range of cognitive deficits, such as slowing in information processing, poor attention and executive function, or poor memory (Sachdev and others 2014).

It is well recognized that although cognitive phenotypes and imaging can indicate one of the prototypical dementias associated with a particular molecular pathology, presentations vary greatly and the syndromes associated with different pathology can mimic each other. Moreover, multipathology is increasingly common as one ages, and someone in their eighties may have up to eight different types of pathology (Robinson and others 2021). Specifically, neuropathologic changes of LBD and AD co-occurred in 50% to 70% of cases in some studies, which makes measuring these proteins important in patients with these conditions (Brenowitz and others 2017; Irwin and others 2017).

The situation is complicated by considering the explanatory gap between neurodegeneration and the associated symptoms. Although accumulated neuropathologic changes are linked to an increased likelihood of dementia in the future, this link is not absolute. Studies of the very elderly have shown that a significant proportion of people aged >90 y have evidence of multiple pathologies in their brains without overt cognitive decline (Kawas and others 2015). One explanation that has been provided is that there are hidden genetic and environmental factors that may give an individual resilience against cognitive decline and dementia (Stern and others 2020). However, there is little understanding what the term *resilience* actually refers to in the brain and in relation to the neurobiology of dementia. This makes it difficult to test claims that sensory loss causes dementia by reducing resilience. With this caveat, we speculate on such a mechanism in the later sections, as this has intuitive appeal and sensory impairment may have a better link with the concept of resilience.

Can a neurobiological understanding of the neurodegenerative markers of dementia allow us to better understand the link with sensory loss? The association of severity of impairment in any sensory modality with a particular pathologic entity would be important, as it could signify that the sensory impairment is a manifestation of the disease process. For example, finding that the severity of olfactory impairment correlates with the stage of AD, as determined by biomarkers, may suggest that AD can manifest as early olfactory impairment. Yet, noticing that biomarkers for several dementias worsen with sensory impairment may suggest a global mechanism that increases risk for any dementia. If sensory loss can cause dementia, which dementia subtype does it affect, and how are the neurobiological processes altered? Studies that aim to shed light on this are still in their infancy. In some sensory modalities, such as olfaction and hearing loss, there are hints that sensory loss is related to certain pathologies, but more work is needed to support these findings (Olofsson and others 2016; Tuwaig and others 2017). The following sections discuss these in detail.

## Does Sensory Loss Cause Dementia?

Epidemiologic studies have linked individual sensory systems to cognitive decline and dementia (Livingston and others 2020; Tran and others 2020; Wheeler and Murphy 2021). In this section, we present evidence for the link between each type of sensory loss and neurodegenerative markers of dementia. We also consider impairments in multiple domains and speculate about the effect that these have on the risk of dementia.

### Olfactory Impairment and Dementia Risk

Olfactory impairment is a well-recognized precursor to the motor symptoms of PD, in which it is present in approximately 90% of early-stage cases, but its direct link with cognitive impairment and dementia has not received as much attention (Devanand and others 2020; Doty 2012). Olfactory dysfunction before death has been described in autopsy-proven cases of LBD with alpha-synuclein deposition in the brain (Driver-Dunckley and others 2014). In the case of LBD, olfactory dysfunction seems to be part of the disease process, despite it preceding symptoms by almost a decade in some cases (Doty 2012). Neuropathologic data demonstrate a relationship between olfactory dysfunction and LBDs, such as PD and dementia with Lewy bodies, which are regarded as a spectrum.

**Figure 1.**
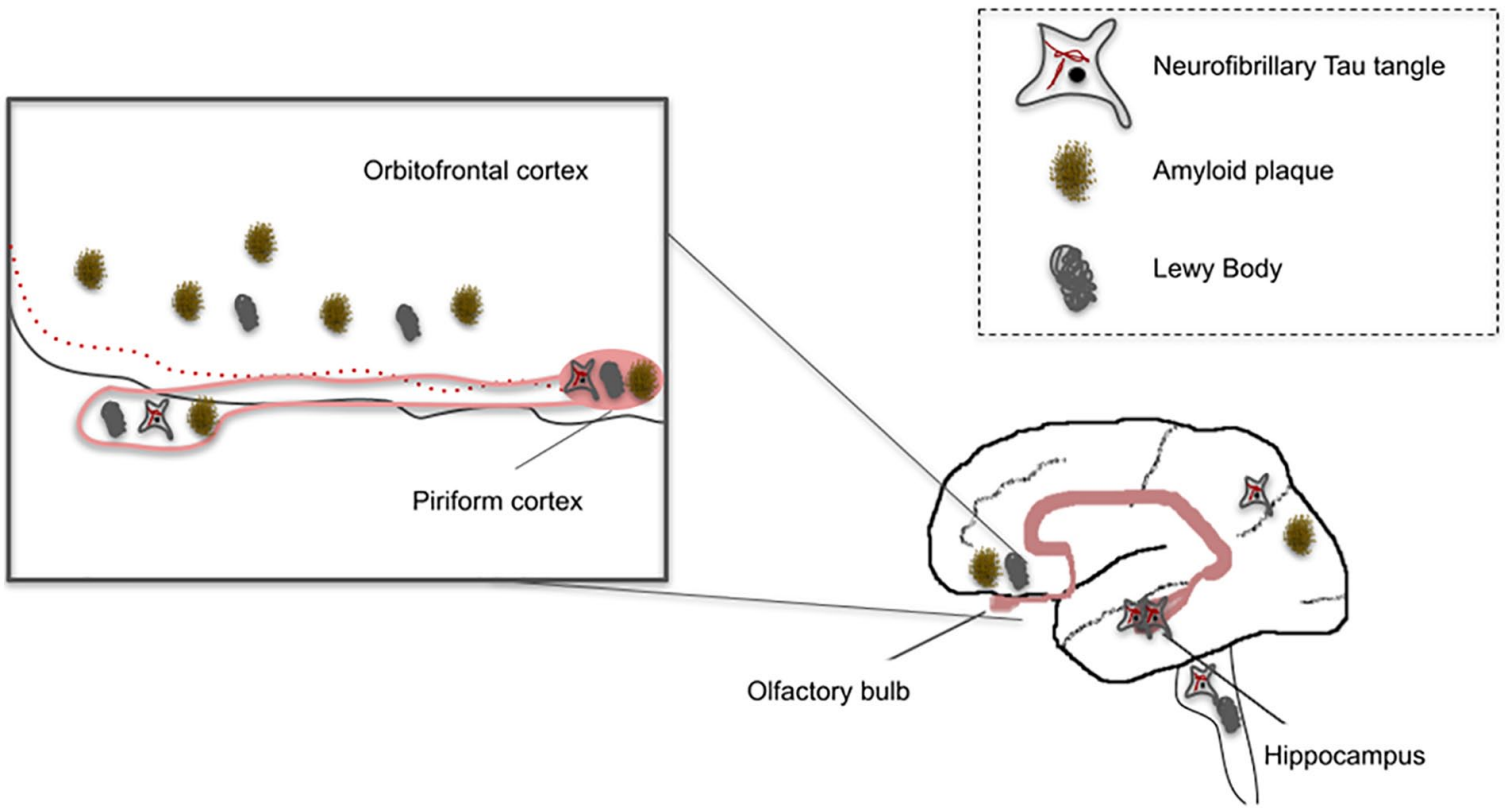
Olfactory system involvement in Alzheimer and Parkinson disease neuropathology is increased in the olfactory bulb with increasing disease severity (Attems and others 2014). The olfactory bulb also has prominent inputs to the hippocampus that are affected early in Alzheimer disease so that odor identification tests might allow the detection of early disease stages. Amyloid, tau, and Lewy body pathology is shown within the olfactory system and in early stages of Alzheimer and Parkinson disease around the brain.

The neural mechanisms of how olfactory dysfunction occurs in LBD is poorly understood, but evidence suggests that the olfactory system may be one of the first sites of neuropathology deposition in the brain of people at risk of LBD-related dementias (Doty 2012) (Figure 1). Lewy body pathology is most likely to occur incidentally in the olfactory bulb, and synucleinopathy density scores in this region are positively correlated with motor symptoms in LBD, suggesting that pathology develops early and continues to develop over time (Driver-Dunckley and others 2014). Other evidence suggests that Lewy pathology may spread from peripheral olfactory areas to central olfactory areas and then to other parts of the brain typically involved in LBD (Rey and others 2018).

An increased risk of cognitive decline and AD dementia has been identified in subjects with olfactory dysfunction. Odor identification has been identified as a useful screening tool that predicts conversion from MCI to AD dementia (Quarmley and others 2017). This finding was replicated in a sample of 728 older community-dwelling individuals, showing that subjects with intact cognition and normal olfactory identification did not transition to dementia after 4 y (Devanand and others 2020). Prediction of the conversion from MCI to AD dementia can be improved by combining information about a person’s *ApoE* genotype, with those with ε4 more likely to convert (Wheeler and Murphy 2021). Poor performance on odor identification tasks has been more broadly linked to a faster rate of cognitive decline in people without dementia (Dintica and others 2019). In this study, there was a faster rate of decline in people with anosmia (complete lack of smell) as compared with those with hyposmia (partial reduction in olfactory function). People with hyposmia still had a faster rate of cognitive decline than those people without any olfactory impairment.

ApoE status, a known genetic risk factor for AD dementia, may modulate the link between olfactory dysfunction and AD. Having the *ApoE4* genotype significantly increases the chances of developing olfactory impairment, cognitive decline, and AD (Misiak and others 2017). Olfactory deficits in carriers of the *ApoE4* genotype also correlate with specific neuropsychological markers of AD severity, such as episodic memory deficits, and neuroimaging markers, such as hippocampal volume (Murphy and others 2003; Olofsson and others 2016). A population-based sample of 1087 people showed that performance on the Scandinavian Odor Identification Test predicted the rapidity of episodic memory decline in people with carriers of the *ApoE4* gene (Olofsson and others 2016). The mechanism for this link is unclear, but animal studies suggest that ApoE has a role in the differentiation and regeneration of olfactory neurons (Nathan and others 2004).

Just as in the case of PD, people with olfactory dysfunction who develop AD show neuropathologic evidence of AD in the olfactory system (Dibattista and others 2020) (Figure 1). Neurofibrillary tangles and Aβ plaques are found in the olfactory bulbs and tracts and in central brain regions that involve memory and olfaction. The olfactory bulbs are affected very early in the AD disease course (Kovács and others 2001). Neuroimaging evidence of disease severity is more apparent in people with olfactory dysfunction. A study with 37 participants showed that left hippocampal volume correlated with performance on an odor identification task (Murphy and others 2003). These studies point to a direct association between olfactory dysfunction and AD neuropathology.

It is possible that the effects of olfactory deficits on dementia may not be direct but via links to areas of the brain involved in cognition (Saive and others 2014). Odors are strong memory cues, and the olfactory system has direct and close anatomic connections to the hippocampus—a key player in the formation and retrieval of episodic memories, which are impaired early in typical AD dementia. Odor identification is strongly related to the amount of semantic information associated with the source and odor-naming ability (Frank and others 2011). Therefore, impairment in an odor identification may signify an impairment in a higher-order cognitive system and not the olfactory perception per se. Additional experiments with different types of olfactory tasks reflecting sensory and cognitive functions, such as discrimination and identification, are needed to clarify the mechanisms for the deficit in AD. Discrimination deficits would occur in a sensory deficit, whereas preserved discrimination with abnormal naming would point to a higher-level deficit.

There is a good case for olfactory deficits being specific risk factors for LBDs and AD and markers of disease severity (Attems and others 2014). Further work is needed to define the deficit in perceptual or cognitive terms and the associated pathology in detail. Special attention is also needed to measure in vivo neurodegenerative disease processes with amyloid, tau, and alpha-synuclein for each person with sensory impairment, owing to the high rate of co-occurrence of neuropathology for both diseases.

### Visual Impairment and Dementia Risk

Visual impairment is estimated to affect 1 billion people by 2050, so the need to clarify whether there is an increased future risk of dementia in the visually impaired is substantial (Bourne and others 2017). Poor vision in later life has been linked with an increased risk of subsequent cognitive decline and dementia (Swenor and others 2019; Tran and others 2020). There are also visual markers of specific disease processes and disease severity that have been recognized (Flanigan and others 2018; Leyland and others 2020). Visual deficits have been examined in association with a variety of age-associated eye diseases, based on various tests for specific kinds of perception and cognitive abilities. These tests probe different neural pathways, and it is unclear how visual impairment in these tests is related to neurodegenerative disease markers and future dementia risk.

Studies have shown that visual impairment (visual acuity <6/12) is more likely to be present in people with dementia living in care homes, with almost 50% being correctable with spectacles and the rest with cataract surgery (Bowen and others 2016). The study noted a range of visual problems, including cataracts, age-related macular degeneration, glaucoma, and diabetic retinopathy. Although the study measured the prevalence of visual impairments only in dementia, at a minimum it highlights the burden of potentially treatable sensory impairment in people with all forms of dementia, after they have a diagnosis. Whether the treatment of these conditions reduces the risk of further cognitive impairment while a person has a dementia diagnosis has not been studied.

Certain measures of visual function are sensitive markers of cognitive dysfunction in PD. Performance on object identification tasks with skewed images and biological motion have been linked to cognitive function in patients with PD (Leyland and others 2020; Zarkali and others 2021). Performance in the former task has been also linked to white matter abnormalities in the brain that may underlie neurodegeneration in PD leading to cognitive decline (Zarkali and others 2021). A study with 52 patients showed that color vision testing is impaired in dementia with Lewy bodies but not AD and correlates with disease severity (Flanigan and others 2018). Further work is necessary to establish whether this finding correlates with neuropathology in vivo, but there are indications that some aspects of visual dysfunction map onto particular diseases, potentially related to alpha-synuclein burden. Studies are needed that track these visual measures in healthy people over time to assess the risk of cognitive decline due to LBD . This would clarify whether certain visual pathways are linked to the underlying neurodegenerative disease process in these conditions.

Some visual measures have been studied in relation to risk of cognitive decline in people without dementia and to their future risk of dementia. Impairments in visual acuity, contrast sensitivity, and stereo acuity are associated with greater declines in scores in the Modified Mini-Mental State Examination, a common screening test for dementia (Swenor and others 2019). A study including 1061 older women from the Women’s Health Initiative Population showed that visual impairment in acuity was associated with a two- to fivefold elevated risk of incident dementia (Tran and others 2020). However, none of these studies provided measures of in vivo neurodegenerative disease.

Other studies have examined whether visual impairment and neuropathology are related postmortem. Animal studies have shown increased AD pathology in the retinas of visually impaired mice (Perez and others 2009). Yet, postmortem histopathologic studies in humans have not shown an increased prevalence of AD pathology in the retinas or brains of subjects with age-related macular degeneration (Schwaber and others 2020). Despite this, there is still interest that retinal pathology may be a biomarker for AD (Ikram and others 2012).

Recent work has shown that treating visual impairment can reduce the future risk of dementia. Treatment of cataracts was associated with a lower risk of incident dementia in a prospective longitudinal cohort study of cognitively unimpaired individuals (Lee and others 2022). The group consisted of 3000 people with a diagnosis of cataracts or glaucoma. Cataract extraction was associated with a reduced hazard ratio (0.71; 95% CI, 0.62–0.83) of developing dementia as compared with participants who did not have surgery, after controlling for years of education, age, sex, race, and ApoE genotype. Glaucoma surgery, which does not improve vision, did not reduce dementia risk. This study provides encouraging data that treating visual impairment can reduce dementia risk, but further studies are needed to replicate this finding and any changes to neurodegenerative markers that might underlie this.

**Figure 2.**
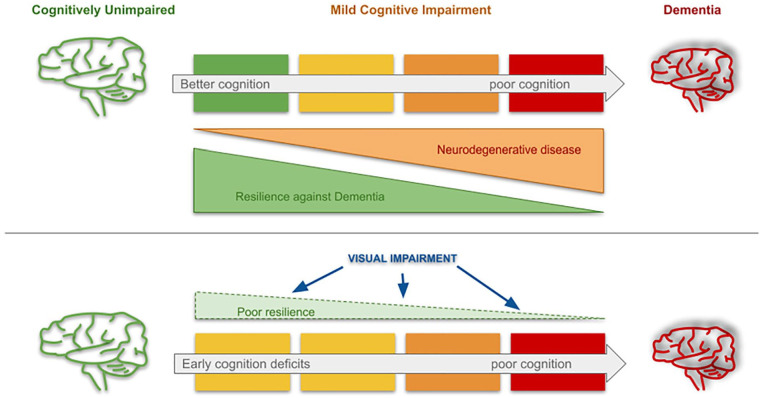
Potential mechanisms relating visual deficits to dementia. Individuals with neurodegenerative disease may lie on a spectrum and show varying cognitive abilities due to their resilience against dementia (upper panel). Visual impairment (lower panel) from primary eye pathology may negatively affect cognitive reserve, thus increasing future risk of dementia from neurodegeneration.

The effect of visual impairment on the future risk of dementia may be indirect. Reduced visual input from the eye can also cause structural changes in and around the primary visual cortex, which recovers on treatment (Hanson and others 2019). Whether these changes alter dementia risk in the future is unclear. Another mechanism by which visual impairment may be related to dementia is via individual resilience (Figure 2). *Resilience* refers to an individual’s ability at any given time to compensate for neuropathologic changes in their brain. People may vary in their resilience or their ability to cope with neuropathologic changes even before visual impairment has set in. In this scenario, visual impairment measures may not directly relate to disease-specific neurodegenerative markers. Treating cataracts could help people with poor resilience and reduce their future risk of dementia. Interestingly, boosting resilience by participating in visual leisure activities has been directly associated with a reduced risk of dementia in cohort studies (Verghese and others 2003).

### Hearing Loss and Dementia Risk

**Figure 3.**
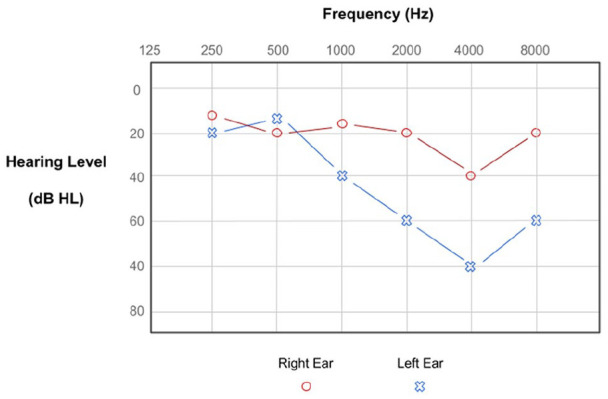
A pure tone audiogram result. The diagram shows the level of amplification in decibels (y-axis) of a pure tone at varying frequencies in hertz (x-axis) for an individual’s left ear (blue crosses) and right ear (red circle). At high frequencies, a greater level of amplification is required for the left ear (moderate hearing loss) than the right ear (mild hearing loss). This pattern of hearing loss is common with aging.

Hearing loss in midlife, measured with PTA (Figure 3), predicts dementia risk 5 to 10 y later. Three cohort studies yielded a combined relative risk of 1.94 for all-cause dementia and a stratification of risk according to the degree of hearing loss (Livingston and others 2020). The strengths of these studies include long follow-up and community-based recruitment of participants. The statistical prediction model cited a number of covariates, such as risk factors for VaD, but did not account for mood or physical activity. The diagnosis of individuals with dementia was also made clinically with no confirmation on biopsy or autopsy (which were the only potential methods for neuropathologic confirmation at the time). There were no neuroimaging, genetic, or biomarker data to gain further mechanistic insights into the pathophysiology of the link between hearing loss and dementia. Nonetheless, these landmark studies provide the best-quality evidence so far for peripheral hearing loss increasing the risk of dementia in the future.

**Figure 4.**
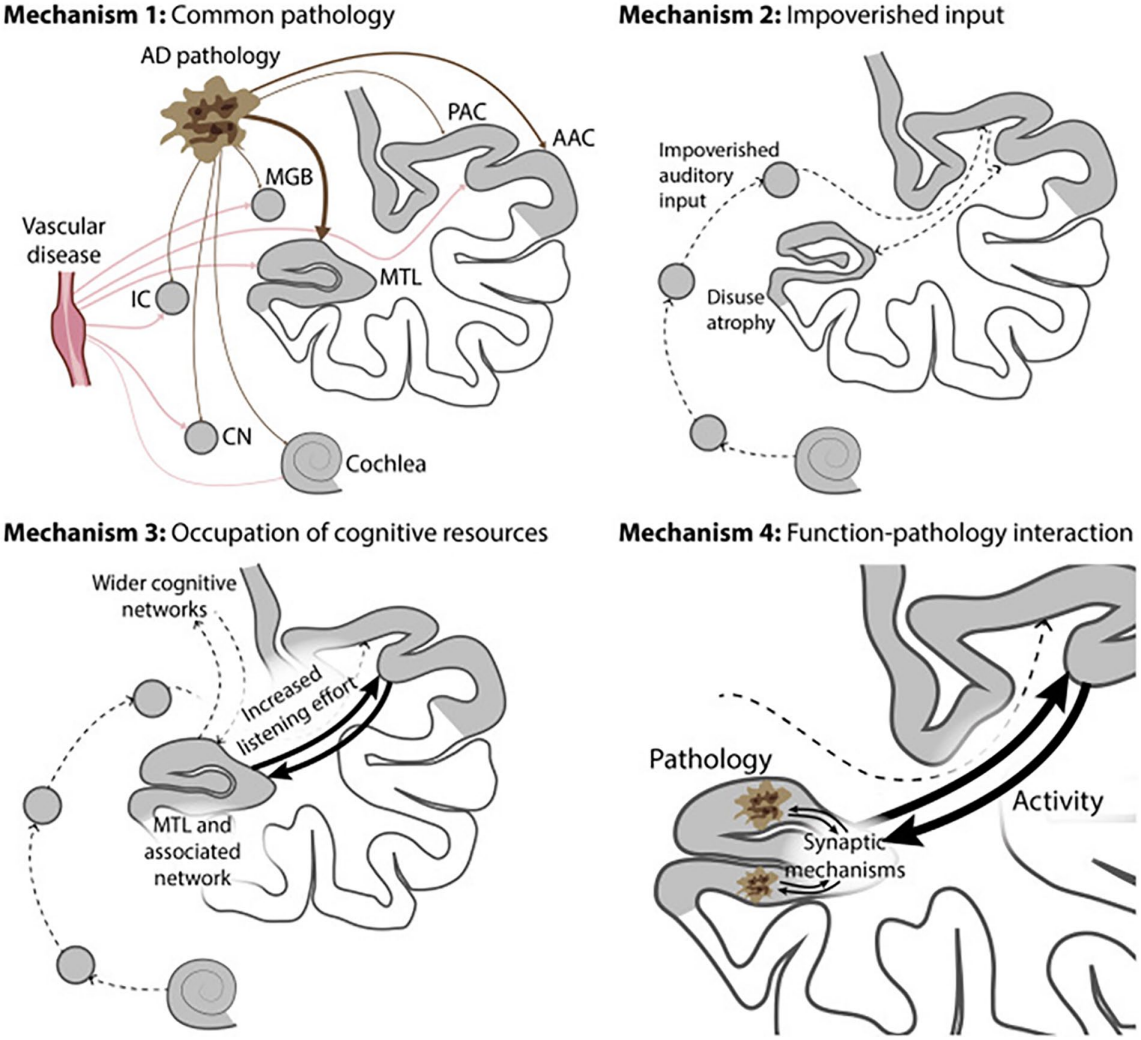
Possible mechanisms for dementia related to hearing loss. Mechanism 1: common pathology due to AD or vascular disease affects the cochlea and/or the ascending pathway (causing hearing loss) and MTL (causing dementia). Mechanism 2: impoverished environment caused by hearing loss leads to altered brain structure in the auditory cortex and hippocampus and decreased cognitive reserve and therefore to decreased resilience to dementia. Mechanism 3: increased brain activity in the MTL and a wider network during speech-in-noise analysis compete for the resources within that network that are needed for other aspects of higher cognition. We argue in the text that this may be a better model for hearing loss–related cognitive deficits in elderly people as opposed to dementia per se. Mechanism 4: interaction between altered activity related to pattern analysis in the MTL during difficult listening and the pathology of AD. The model is based on the same mechanism for increased activity as mechanism 3, but it differs in the incorporation of a specific interaction with the molecular bases of AD. This is based on an interaction between increased activity and synaptic changes associated with AD. We also consider a mechanism in the text due to decreased activity interacting with AD pathology (not shown here). AAC = auditory association cortex; AD = Alzheimer disease; CN = cochlear nucleus; IC = inferior colliculus; MGB = medial geniculate body; MTL = medial temporal lobe; PAC = primary auditory cortex. Reproduced using the CC BY 4.0 licence (Griffiths and others 2020).

We previously discussed the mechanisms by which hearing loss may be related to dementia (Griffiths and others 2020) (Figure 4). These include neuropathology in the ascending auditory pathway and cortex, poor cognitive reserve due to an impoverished listening environment, occupation of cognitive resources in difficult listening environments, and pathology in the medial temporal lobe interacting with central auditory pathways. The mechanisms may overlap and the contributions of each could vary in an individual. Currently the links between hearing loss and dementia are based on links between the PTA and a clinical diagnosis of dementia where major categories include AD and vascular dementia based on clinical criteria only. It is important to note that many of the cohort studies that identified the link between hearing loss and dementia statistically controlled for cardiovascular risk factors, which also predict VaD, and so this may suggest that the risk is driven by nonvascular pathologies.

More data are required on the link between hearing loss and the neurodegenerative disease markers of specific dementias. The epidemiologic studies used to date do not include biochemical and neuroimaging biomarkers that predict the likelihood of a specific dementia syndrome in the form of amyloid, tau status, alpha-synuclein, and vascular neuroimaging burden. One cross-sectional study showed that PTA thresholds did not correlate with brain amyloid deposition, hippocampal volumes, or VaD burden (Parker and others 2019). However, measuring hearing loss and dementia biomarkers at the same time may not capture the risk, due to hearing loss over the life course. Studies following individuals over time have found hearing loss to be associated with reduced hippocampal volumes (Lin and others 2014). One cross-sectional study determined that central auditory tests, such as speech-in-noise perception abilities, correlated with raised tau in the cerebrospinal fluid and lower medial temporal lobe volumes, after adjusting for age, sex, education, PTA thresholds, and *APOE* status (Tuwaig and others 2017). This finding suggests central auditory dysfunction to be a more proximate indicator of AD than peripheral measures such as the PTA, but longitudinal studies are needed for corroboration.

There is a growing body of literature that the medial temporal lobe including the hippocampus, key brain structures implicated in AD, is involved in auditory processing (Billig and others 2022). Human functional imaging studies demonstrate the involvement of these structures in real-world listening under adverse conditions, such as speech-in-noise perception (Griffiths and others 2020). Auditory cognition relevant to real-world listening, including working memory for sounds, has been shown to depend on hippocampal mechanisms (Billig and others 2022). Rodent work supports specific roles for medial temporal lobe structures in auditory cognition: Aronov and others (2017) demonstrated that single units in the hippocampus were tuned to specific frequencies during a working memory task, consistent with the use of hippocampal computational mechanisms for auditory analysis. We have suggested that increased activity in medial temporal lobe mechanisms for auditory cognition during real-world listening, acting to compensate for peripheral hearing loss, might augment the early pathologic mechanisms for AD in the same areas (Griffiths and others 2020) (Figure 4). Other rodent work has shown that increased external auditory stimulation improves spatial cognition and decreases amyloid deposition in the auditory cortex and hippocampus in a genetic mouse model of AD (Martorell and others 2019). Future work is needed to understand the links among the degree of ongoing auditory stimulation, its neuropathologic effects, and the risk of dementia. Additionally, although evidence links hearing loss to AD, it may add LBD, as the co-occurrence of these pathologies is high. Therefore, there is a case for investigating hearing loss in other dementias as well.

### Multisensory Impairment and Dementia

A small number of studies have examined the co-occurrence of impairment in multiple sensory domains and the risk of subsequent cognitive decline or dementia. The Blue Mountains Eye Study, involving thousands of people, studied the impact of reduction in visual acuity scores and hearing loss on Mini-Mental State Examination scores over a 15-y period and found that neither visual impairment nor hearing loss or dual sensory impairment was independently associated with subsequent decline in cognition (Hong and others 2016). A study of an aging cohort reported that combined visual and auditory sensory impairment was related to faster cognitive decline than unisensory impairment (Parada and others 2021). Poor multisensory composite scores—composed of objective measures of visual acuity, smell identification, touch vibration detection threshold, and audiometric assessments—were recently associated with an increased risk of all-cause dementia (Brenowitz and others 2020). Finally, a study determined that low self-reported hearing and vision scores were related to a higher risk of all-cause dementia and AD in particular (Hwang and others 2020).

Elucidating the basis for the association between multisensory impairment and dementia risk is a challenge, as impairment from one domain may interact with impairment in another (Brenowitz and others 2020). Another challenge is that sensory impairments may worsen preexisting mobility, depression, and social isolation, which have independent effects on cognitive decline and dementia (Fischer and others 2009). Additional studies need to study these factors thoroughly alongside multisensory impairments to estimate the impact of each on future dementia risk. Some have expressed concerns that such impairments may lead to measurement error and an overestimation of the cognitive deficit of sensory impaired individuals, so caution is required when assessing the findings of all of these studies (Jorgensen and others 2016; Toner and others 2012). Further studies need to use validated and sense-suited cognitive tests for those with sensory impairments.

The aforementioned studies have assessed well-defined cohorts with pure sensory impairments and suggest links between sensory loss and dementia in olfaction, vision, and hearing. More work is required to move beyond observational clinical links between sensory loss and dementia syndromes, to establishing possible specific neuropathologic bases for these interactions related to certain neurodegenerative disease processes. In reality, the examination of individual deficits might be considered an artifice. An aging person may gradually accumulate a reduction of visual acuity, hearing, olfaction, and cognition along with other things. Also, people may cope with their sensory impairment to varying degrees, and some may be susceptible to mood disorders or social isolation, which has an independent effect on cognition and dementia risk (Livingston and others 2020). There is an additional assumption in many of the studies presented here that cognitive decline or dementia risk is driven by a “bottom-up” process, whereby sensory impairment is causative of subsequent cognitive decline. However, there is increasing recognition of the role that “top-down” brain processes play even in the perceptual tasks used to measure performance in a particular sensory domain (Kocagoncu and others 2021).

### Common Convergent Sensory Pathways to Dementia or Preferential Risk Determined by One Sensory Modality?

Olfaction, vision, and hearing have unique ascending inputs to the brain for perception, which converge at higher levels. Currently, it is unclear whether the future risk of dementia from sensory impairment is a result of neural pathways that are unique for each sensory modality or the higher-level pathways in the brain that may not be modality specific. The respective primary cortices are present in the inferior frontal, lateral temporal, and occipital poles, but these areas have well-described inputs to other cortical and subcortical areas. As described earlier, olfactory and visual areas show some overlap with disease stages and progression in AD and PD in its course, whereas this is less clear-cut in visual areas (Dibattista and others 2020; Doty 2012; Flanigan and others 2018). A rare variant of AD dementia, posterior cortical atrophy, however, does present with early deficits in higher-order visual processing due to abnormal protein accumulation in these regions.

In commoner dementia types, the absence of neuropathology in the brain regions associated with a particular sense may not indicate the absence of additional risk of dementia, as functional interactions could be at play (Griffiths and others 2020). For example, the auditory system exhibits the phenomenon of central gain enhancement to reduced peripheral input. Central brain structures increase their activity to sound due to reduced input (Auerbach and others 2014). However, the functional implications of such gain enhancement to downstream structures such as the hippocampus are unknown. One candidate is that changes in central gain affect attentional processes that subserve other cognitive domains. It is possible that central gain changes through hearing loss affect changes in central gain that are possible through attentional changes (Auerbach and Gritton 2022). The discussion of these factors is beyond the scope of this review. How this functional change then affects other cognitive processes related to hearing and in general is not known.

**Figure 5.**
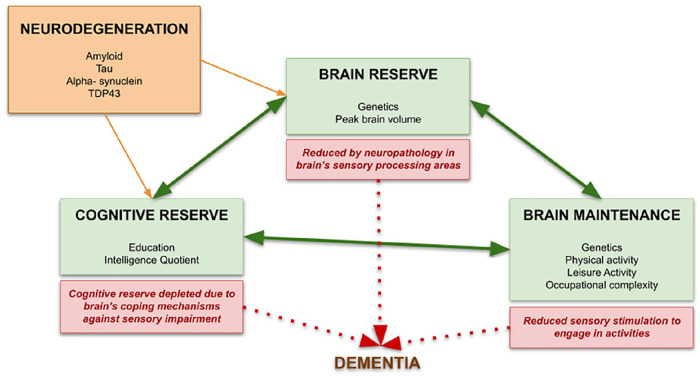
The effect of sensory impairment on resilience to dementia. Three factors may make up an individual’s resilience to dementia: *brain reserve*, the anatomic and structural characteristics of the brain protecting against toxic neuropathologic effects; *cognitive reserve*, the adaptability of cognitive processes that explains interindividual susceptibility to brain aging, pathology, or insult; and *brain maintenance*, the reduced development of age-related or pathologic brain changes over time. The green arrows indicate that these domains may interact and are not mutually exclusive. Sensory impairment, from olfactory, hearing, or visual impairment, may increase dementia risk by acting on one or more levels. Neuropathology in areas of the brain supporting sensation can directly lower brain reserve through toxic effects. Sensory impairment may divert resources from a person’s cognitive reserve to help cope with its effects. Reduced sensory input and stimulation over time may also reduce activities promoting healthy brain maintenance. All of these effects could in turn increase a person’s risk for dementia (as indicated by the dashed red arrows).

Many studies have indicated that some individuals are more resilient to dementia than others, despite having the same risk factors (Stern and others 2020). They may have protective factors in their brain structure, cognitive abilities, or factors that help maintain a healthy brain. These concepts have only recently emerged, but research in this area is expanding. Sensory impairment may have an impact on the brain through any of these mechanisms (Figure 5). There is evidence that high metabolic activity in some brain regions is linked to a lower rate of cognitive decline (Arenaza-Urquijo and others 2019). These include the anterior cingulate cortex and anterior temporal pole: areas of the brain implicated in a range of tasks and diseases (Herlin and others 2021; Rolls 2019). These areas may serve as multimodal processing areas that can be affected by impairment in any sensory modality. Further work is needed to establish whether sensory impairment can modulate the signatures of cognitive resilience that have been identified with respect to dementia

### Future Directions

As the emphasis of dementia prevention and diagnosis moves earlier in the life course, there is more interest in identifying clinical or biological markers that put an individual at risk of developing dementia (Livingston and others 2020). In parallel, there are efforts to identify clinically applicable neurodegenerative disease-specific markers in preclinical stages. The greatest advance in the latter has occurred with AD where certain blood markers can predict dementia due to AD with a very high degree of fidelity (Palmqvist and others 2021). A combination of such markers can help categorize non–AD-related disease processes (Chouliaras and others 2022). Future work needs studies where sensory markers are prospectively combined with neurodegenerative markers to see if impairment in a sensory domain leads to elevations in disease-specific biochemical markers (Table 1). This will help establish whether treatment of sensory impairment can be disease modifying. Special consideration must be given to measure a range of markers together. For example, measuring AD and LBD markers may be prudent owing to the high rates of copathology.

**Table 1.** Gaps in Knowledge Linking Sensory Loss in Olfaction, Vision, and Hearing and Future Risk of Dementia.

	Sensory Modality		
	Olfaction	Vision	Hearing
AD			
Epidemiologic	+	?	+
Neuroimaging	–	–	+
Neurodegenerative	+	–	+
LBD			
Epidemiologic	+	?	–
Neuroimaging	+	–	–
Neurodegenerative	+	–	–
VaD			
Epidemiologic	–	?	+
Neuroimaging	–	–	–
Neurodegenerative	–	–	–
FTD			
Epidemiologic	–	?	–
Neuroimaging	–	–	–
Neurodegenerative	–	–	–
Other dementias: LATE, AGD	–	–	–

The presence of evidence is indicated by a plus (+), whereas the lack of evidence is shown by a minus (–). A question mark (?) indicates uncertainty regarding a link, as studies for these links have not specified a dementia subtype. AD = Alzheimer disease; AGD = argyrophilic grain disease; FTD = frontotemporal dementia; LBD = Lewy body disease; LATE = limbic-associated TDP-43 encephalopathy; VaD = vascular disease.

Hearing aids and treatment of some forms of visual impairment are perhaps the only methods by which sensory perception can be improved or aided. There are trials underway to see whether treating hearing impairment with hearing aids can slow or reverse cognitive decline (Deal and others 2018). The results of these are eagerly awaited. There remain practical issues with assessing compliance in people who use hearing aids, so another potential patient group that may yield insight into these questions is people who undergo cochlear implantation after surgery (Sarant and others 2019). In vision, it is also unclear how cataract treatment may reduce risk of subsequent dementia (Lee and others 2022). If there are clear changes to neurobiological markers of neurodegeneration, then there is cause for optimism for a nonpharmacologic disease-modifying agent in the future.
